# Analysis of clinical *Candida parapsilosis* isolates reveals copy number variation in key fluconazole resistance genes

**DOI:** 10.1128/aac.01619-23

**Published:** 2024-05-07

**Authors:** Sean Bergin, Laura A. Doorley, Jeffrey M. Rybak, Kenneth H. Wolfe, Geraldine Butler, Christina A. Cuomo, P. David Rogers

**Affiliations:** 1 School of Biomolecular and Biomedical Science, Conway Institute, University College Dublin, Belfield, Dublin, Ireland; 2 Department of Pharmacy and Pharmaceutical Sciences, St. Jude Children’s Research Hospital, Memphis, Tennessee, USA; 3 School of Medicine, Conway Institute, University College Dublin, Belfield, Dublin, Ireland; 4 Infectious Disease and Microbiome Program, Broad Institute of MIT and Harvard, Cambridge, Massachusetts, USA; 5 Molecular Microbiology and Immunology Department, Brown University, Providence, Rhode Island, USA; University of Iowa, Iowa City, Iowa, USA

**Keywords:** azoles, genomics, CNV, yeasts, drug resistance mechanisms

## Abstract

We used whole-genome sequencing to analyze a collection of 35 fluconazole-resistant and 7 susceptible *Candida parapsilosis* isolates together with coverage analysis and GWAS techniques to identify new mechanisms of fluconazole resistance. Phylogenetic analysis shows that although the collection is diverse, two persistent clinical lineages were identified. We identified copy number variation (CNV) of two genes, *ERG11* and *CDR1B*, in resistant isolates. Two strains have a CNV at the *ERG11* locus; the entire ORF is amplified in one, and only the promoter region is amplified in the other. We show that the annotated telomeric gene *CDR1B* is actually an artifactual *in silico* fusion of two highly similar neighboring *CDR* genes due to an assembly error in the *C. parapsilosis* CDC317 reference genome. We report highly variable copy numbers of the *CDR1B* region across the collection. Several strains have increased the expansion of the two genes into a tandem array of new chimeric genes. Other strains have experienced a deletion between the two genes creating a single gene with a reciprocal chimerism. We find translocations, duplications, and gene conversion across the *CDR* gene family in the *C. parapsilosis* species complex, showing that it is a highly dynamic family.

## INTRODUCTION

*Candida parapsilosis* is a human fungal pathogen that is globally one of the most common sources of non-*albicans Candida* infections (1, 2). In the decade 2006–2016, *C. parapsilosis* accounted for ~16% of all candidemia cases (3). Traditionally, *C. parapsilosis* was predominantly found in immunocompromised patients such as transplant recipients or preterm neonates (4, 5). More recently, however, there has been an increase in cases in adult patients in non-surgical wards (6, 7). *C. parapsilosis*, and its sister species *Candida orthopsilosis* and *Candida metapsilosis*, belongs to the CUG-Ser1 clade, along with other major fungal pathogens *Candida albicans*, *Candida dubliniensis*, and *Candida tropicalis* (8). Unlike its sister species and other members of this clade, *C. parapsilosis* is assumed to be completely asexual due to its high homozygosity, pseudogenization of *MAT*a, and the lack of a *MAT*α idiomorph (9, 10).

Outbreaks of *C. parapsilosis* have been associated with variants conferring resistance to common antifungal drugs, including fluconazole, a triazole (11). Fluconazole binds to the enzyme lanosterol 14alpha-demethylase, encoded by the gene *ERG11*. This enzyme plays a key role in the ergosterol biosynthesis pathway, which is inhibited by the binding of fluconazole (12–14). Ergosterol is a key component of the fungal cell membrane, and in its absence, and with accumulation of alternate sterols, cell growth is arrested (15, 16). Resistance to fluconazole treatment is a growing trend in clinical *Candida spp*. isolates (17). In *C. parapsilosis*, resistance is particularly associated with the Y132F substitution in *ERG11* that contributes directly to resistance (18) and has been implicated in many fluconazole-resistant outbreak events across the world (19–24). Equivalent mutations also contribute to fluconazole resistance in *C. albicans*, *C. tropicalis,* and *Candida auris* (25–29).

Most of our understanding of other mechanisms of fluconazole resistance in *Candida* species, including the role of other substitutions in *ERG11*, comes from studies in *C. albicans* (13, 14, 26, 29). Overexpression of *ERG11*, often by gain-of-function mutations in the transcriptional regulator *UPC2*, has been implicated in resistance in *C. albicans* (30–32). In addition, azole resistance is due in part to overexpression of drug efflux pumps (33–35). In *C. albicans*, the contribution of two drug efflux pumps encoded by *CDR1* and *CDR2* both belong to the ABC transporter (CDR) family to fluconazole resistance has been well studied (33). In the absence of drugs, *CDR1* is expressed while *CDR2* is not (36). However, expression of both genes is upregulated in some resistant strains due to activating mutations in *TAC1*, a gene encoding a transcriptional regulator (18, 37, 38). Likewise, *MDR1*, which encodes a transporter of the Major Facilitator Superfamily, is overexpressed in some isolates due to activating mutations in *MRR1*, encoding another transcriptional regulator (39). Overexpression of homologs of *CDR1* and *MDR1* has also been found to contribute to resistance in some *C. parapsilosis* clinical isolates that contain similar activating mutations in *TAC1* and *MRR1* (18, 40, 41). Often, multiple resistance mechanisms are found to act in concert in the same isolate leading to high-level resistance (42, 43).

In this study, we investigate the genetic mechanisms underlying fluconazole resistance in 42 *C*. *parapsilosis* isolates. Fluconazole resistance has previously been studied in 34 of these isolates using targeted gene sequencing and gene expression analysis (18, 40, 41). Mutations in *ERG11* and over-expression of drug transporters were identified in some isolates. However, some isolates that share the same azole resistance-associated mutation exhibit a range of minimum inhibitory concentration (MIC) values, and for other isolates, no obvious resistance mechanisms were identified. Here, we use whole-genome sequencing, coverage analysis, and GWAS methods to identify point mutations and copy number variants (CNVs) associated with novel mechanisms of fluconazole resistance. Using phylogenomic methods, we also identified two persistent lineages from clinics in Bloemfontein and Johannesburg, South Africa.

## RESULTS

### Phylogeny and two persistent lineages

**Fig 1:**
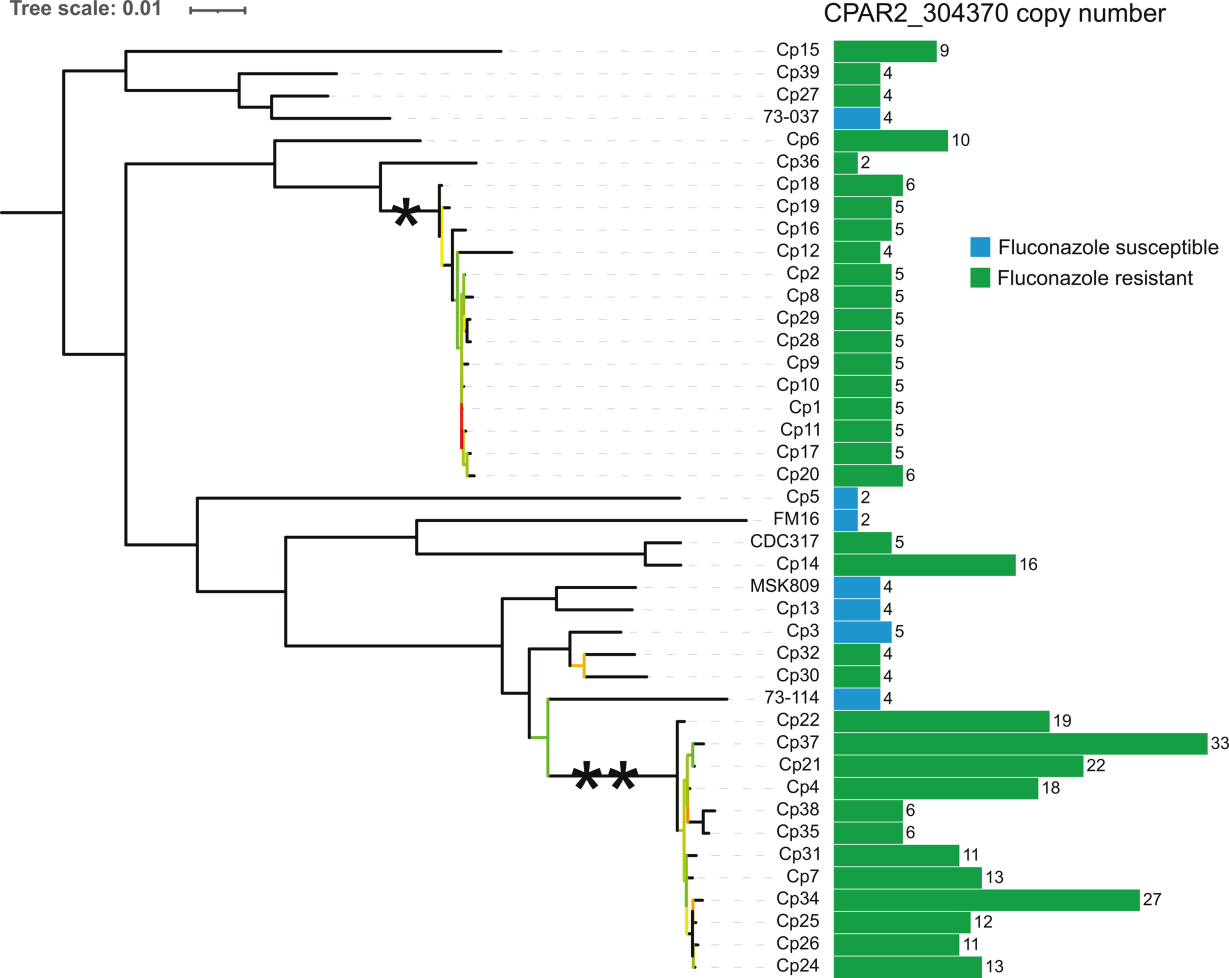
(Left) Maximum-likelihood tree of 42 *C. parapsilosis* isolates. RAxML (45) was used to construct the tree from an alignment of 15,582 SNPs genome-wide, using the GTRGAMMA model of nucleotide substitution. Branches with bootstrap values <100 after 1,000 iterations of bootstrap sampling are colored according to value, ranging from red (0) to green (99). Clades are marked with asterisks to denote persistent lineages in Bloemfontein (*) and Johannesburg (**). (Right) Bar chart showing the estimated copy number of the *CDR1B* locus (*CPAR2_304370* in the reference genome assembly). Fluconazole-susceptible isolates are denoted with a blue bar, and resistant isolates are denoted with a green bar. The copy number was estimated by taking mean coverage across the *CPAR2_304370* ORF and dividing by half the modal coverage for the isolate.

Azole resistance mechanisms have previously been studied in 34 fluconazole-resistant (MIC ≥ 8 µg/mL) and 3 fluconazole-sensitive (MIC ≤ 2 µg/mL) isolates of *C. parapsilosis* included in this study (18, 40, 41). To improve the power of the analysis (especially for GWAS), we sequenced all 37 genomes and included one more resistant isolate CDC317 (the reference strain for *C. parapsilosis*) and four susceptible isolates 73/037, 73/114, FM16, and MSK809 (44). The isolates originate from several geographical locations, including several collected from two cities in South Africa between 2001 and 2009 (Johannesburg and Bloemfontein; Table 1). Phylogenomic analysis shows that the isolates represent a broad range of the *C. parapsilosis* phylogeny, as seen when integrated into a tree containing >200 other strains (Fig. S1) (44). Resistant isolates fall into each of the five global clades of *C. parapsilosis* that we have previously identified (44), and susceptible isolates belong to four out of five clades. Despite this breadth, two groups of isolates have very shallow branches, indicating that they have a very close relationship (Fig. 1). The clade marked by a single asterisk contains isolates all originating from the same clinic in Bloemfontein (Table 1). For the clade marked with double asterisks, one isolate comes from Ann Arbor, Michigan, whereas the rest originate from a clinic in Johannesburg, collected over a period of 8 years. These clades likely indicate *C. parapsilosis* lineages that have persisted in South African clinics (and possibly in the environment) over 8 years.

**TABLE 1:** Expression and variant analysis of *C. parapsilosis* isolates

Isolate	Origin	Date M/Y	*^a^*MIC	Expression relative to Cp13 (log_2_FC)*^b^*					CNV CDR1B	*^a^*Variants		
				ERG11	MDR1	MDR1B	CDR1	CDR1B		ERG11	MRR1	TAC1
Cp37	Johannesburg	07/08	256	**0.53**	0.89	0.25	−0.45	**1.84**	33	**Y132F, R398I**		L978W
Cp38	Johannesburg	08/08	32	0.11	0.6	−0.31	**0.73**	**2.03**	6	**Y132F, R398I**		**G650E**
Cp35	Johannesburg	01/08	32	0.38	0.78	0	0.37	**1.48**	6	**Y132F, R398I**		**G650E**
Cp34	Johannesburg	03/08	32	−0.07	0.48	0.91	−0.29	**1.65**	27	**Y132F, R398I**		
Cp26	Johannesburg	05/04	32	0.12	0.46	−0.25	−0.23	**1.15**	11	**Y132F, R398I**		
Cp22	Johannesburg	07/03	32	0.25	0.6	0.08	−0.36	**1.26**	19	**Y132F, R398I**		
Cp31	Johannesburg	07/06	32	0.27	0.35	0.16	−0.17	**1.23**	11	**Y132F, R398I**		
Cp24	Johannesburg	09/2003	16	−0.1	0.2	0.05	−0.07	**0.83**	13	**Y132F, R398I**		
Cp25	Johannesburg	09/2003	16	0.12	0.51	0.03	−0.41	**1.24**	12	**Y132F, R398I**		
Cp4	Johannesburg	05/2001	16	**0.82**	**1.01**	**1.47**	−0.15	**1.65**	18	Y132F, **R398I**		
Cp7	Johannesburg	05/02	16	**0.63**	**1.44**	**2.58**	0.09	**1.75**	13	**Y132F, R398I**		
Cp21	Ann Arbor	05/03	8	0.35	0.69	0.3	−0.43	**1.77**	22	**Y132F, R398I**		L978W
Cp28	Bloemfontein	02/05	64	0.34	−0.21	**3.63**	0.2	0.48	5		A854V	
Cp29	Bloemfontein	02/05	64	0.11	**2.53**	**5.28**	−0.22	**2.18**	5		**A854V**	
Cp20	Bloemfontein	04/03	16	0.22	−0.04	**3.94**	**0.59**	**0.94**	6		A854V	
Cp11	Bloemfontein	03/02	16	−0.19	−0.02	**4.56**	−0.08	−0.17	5			
Cp2	Bloemfontein	05/01	16	**0.48**	**1.41**	**4.41**	0.36	**1.25**	5		A854V	
Cp1	Bloemfontein	03/01	16	**0.57**	0.21	**3.65**	0.23	**0.98**	5		A854V	
Cp18	Bloemfontein	04/03	16	**0.5**	−0.18	**2.31**	0.4	0.07	6			
Cp17	Bloemfontein	01/03	16	0.14	−0.07	**1.76**	**−0.6**	−0.01	5			
Cp8	Bloemfontein	01/02	16	**0.72**	**1.46**	**4.13**	0.25	0.39	5			
Cp10	Bloemfontein	03/02	16	0.45	0.45	**4.55**	0.26	**1.03**	5		A854V	
Cp12	Bloemfontein	07/02	16	**−0.68**	**0.9**	**2.28**	−0.4	0.15	4		P255L, A854V	
Cp9	Bloemfontein	03/02	16	−0.29	0.45	**2.18**	**−0.89**	−0.6	5		A854V	
Cp16	Bloemfontein	01/03	8	0.37	−0.29	**3.44**	0.39	−0.12	5			
Cp19	Bloemfontein	04/03	8	**−0.72**	**−0.93**	**1.8**	−0.22	−0.69	5			
Cp30	Bratislava	09/05	128	−0.18	**5.14**	**7.38**	**−0.67**	**3.39**	4	**R398I**	**R479K**	
Cp32	Caracas	06/07	128	**0.92**	0.48	−0.69	0.09	−0.08	4	**R398I**		I221T
Cp36	Detroit	04/08	64	**−0.69**	**4.09**	**6.71**	**−0.53**	**3.11**	2		**I283R**	
Cp39	Bratislava	07/09	32	**0.75**	0.33	**3.26**	−0.43	**1**	4	**Y132F**	G294E	**R208G**
Cp27	Hershey	11/04	32	**2.45**	0.25	**2.7**	−0.06	**2.01**	4		K129fs, G982R	**R208G**
Cp14	Helsinki	06/02	16	0.08	−0.47	0.28	0.42	**2.09**	16			
Cp15	Quito	01/03	16	**2.38**	−0.26	0.58	−0.01	0.17	9	**F145L**		**N900D**
Cp6	Turino	05/01	16	**0.93**	0.45	0.8	**−0.92**	**0.94**	10			
Cp5	New York	N/A	0.5	0.42	0.54	0.7	**−1.26**	−0.57	2		**K177N**, Q1053*	
Cp3	Kuala Lumpur	04/01	0.25	**0.8**	0.25	0.26	−0.14	**0.82**	5	**R398I**		
Cp13	New York	N/A	0.25	0	0	0	0	0	4	**R398I**, S216L		
CDC317	Mississippi	N/A	64*^c^*	–	–	–	–	–	5	Y132F		
FM16	Nantes	N/A	2*^c^*	–	–	–	–	–	2			
MSK809	New York	N/A	2*^c^*	–	–	–	–	–	4	**R398I**		
73–037	Leeds	N/A	2*^c^*	–	–	–	–	–	4			**R208G**
73–114	Leeds	N/A	2*^c^*	–	–	–	–	–	4	**R398I**	D615G	**L877P**

^*a*^ Fluconazole MIC values (µg/mL, 24 h) and variants in *ERG11*, *MMR1*, and *TAC1* were described previously (18, 41). Underlined mutations have been experimentally determined to increase fluconazole resistance. Homozygous variants are shown in bold.

^*b*^ Expression values show log_2_ fold-changes relative to Cp13 from RNA-seq. Expression values in bold have *P*-values < 0.05 (Table S1). Dashes indicate that expression data are not available.

^*c*^ MIC values were measured in a separate assay.

For 34 of the fluconazole-resistant isolates, multiple potential resistance mechanisms were previously identified using gene expression analysis (RT-qPCR) and targeted gene sequencing (18, 40, 41) (Table 1). To improve the resolution, we measured the expression of target genes using RNA-seq, in comparison to the expression of the genes in the azole-susceptible isolate Cp13 (Table 1; Table S1). Principal component analysis (PCA) using this data shows that isolates from the South African clades are more closely related than isolates outside these clades (Fig. S2). The RNA-seq data support the previous expression analysis (40, 41). For example, the drug transporter *CPAR2_603010* (*MDR1B*) is overexpressed in strains from Bloemfontein (log_2_FC ranging from 1.8 to 5.3; Table 1), mediated at least in part by the A854V activating mutation in the regulator gene *CPAR2_807270* (*MRR1*), which contributes directly to resistance to fluconazole (41). Some Bloemfontein strains have increased expression of *MDR1B* but do not have a corresponding *MRR1* mutation (e.g., Cp11; Table 1) (41). Strikingly, the isolate homozygous for A854V, Cp29, has much higher expression of both *MDR1B* (log_2_FC = 5.28) and *CDR1B* (log_2_FC = 2.18) compared to the other Bloemfontein strains. In addition, mutations in the ergosterol biosynthesis gene *CPAR2_303740* (*ERG11*) and the *CDR*-family regulator *CPAR2_303510* (*TAC1*) were shown to contribute to fluconazole resistance in other isolates (40).

Fourteen strains (including the reference strain *C. parapsilosis* CDC317) harbor the *ERG11* Y132F substitution which is a well-documented resistance mutation (17, 18, 25, 40, 46). The Y132F substitution is heterozygous in CDC317 and Cp4 and homozygous in the other 12. The isolates from Johannesburg all have the Y132F mutation (including the heterozygous Cp4), but the isolates from Bloemfontein do not (Table 1).

Eleven of the 35 resistant strains do not have any mutations in *ERG11*, *TAC1*, or *MRR1* that have been experimentally determined to affect fluconazole resistance (Table 1; Cp6, Cp8, Cp11, Cp14, Cp15, Cp16, Cp17, Cp18, Cp19, Cp27, and Cp32). The R398I mutation in *ERG11* present in several isolates (e.g., Cp32) has been frequently observed occurring in tandem with Y132F (21, 24) but has also been found without Y132F in susceptible isolates (40). The substitutions A854V, R479K, and I283R in *MRR1* have all been identified as activating mutations leading to the upregulation of genes including *CDR1B* and *MDR1B* (47). The A854V mutation is found in several members of the Bloemfontein clade, while R479K and I283R are found only in single strains (Cp30 and Cp36, respectively; Table 1). The *TAC1* G650E mutation has been shown to increase fluconazole resistance and overexpress *CDR1* and *CDR1B* when introduced into a susceptible isolate (18). In addition, among this collection of isolates, strains that share the same mutation can differ 32-fold in their MIC values (e.g., isolates in the Johannesburg clade have MICs varying from 8 to 256 µg/mL; Table 1). This suggests that novel resistance mechanisms remain to be identified, and different mechanisms may have additive effects that have not been captured by these studies.

### Copy number variation of *ERG11*

We first analyzed the genomes of all strains to identify CNVs that could contribute to resistance (see Materials and methods). We found that aneuploidy is relatively common in the 42 isolates; 13 have either segmental or whole chromosome aneuploidies, and several isolates have aneuploidies of multiple chromosomes (Fig. S3). There are also several unique stair-step CNVs that span multiple genes including a CNV on chromosome 3 that amplifies 31 genes in Cp22 and Cp24, a CNV on chromosome 8 that amplifies 115 genes in Cp19, and a CNV on chromosome 1 that amplifies 61 genes in Cp16 (Fig. S3). All CNVs are listed in Table S2. Unlike in *C. albicans* where aneuploidy of the chromosome containing *ERG11* and *TAC1* has been associated with fluconazole resistance (48–50), none of the resistant strains in the collection have extra copies of chromosome 3. A single susceptible isolate, Cp3, has three copies of chromosome 3 and also increased expression of *ERG11* (log_2_FC = 0.8; Table 1). Several resistant strains have aneuploidy of chromosome 5, which contains the *ERG4* gene (Fig. S3). The potential role of *ERG4* in fluconazole resistance in *Candida spp*. has not been well characterized, but the gene is overexpressed (as well as *ERG11*) in some azole-resistant *C. albicans* (51).

**Fig 2:**
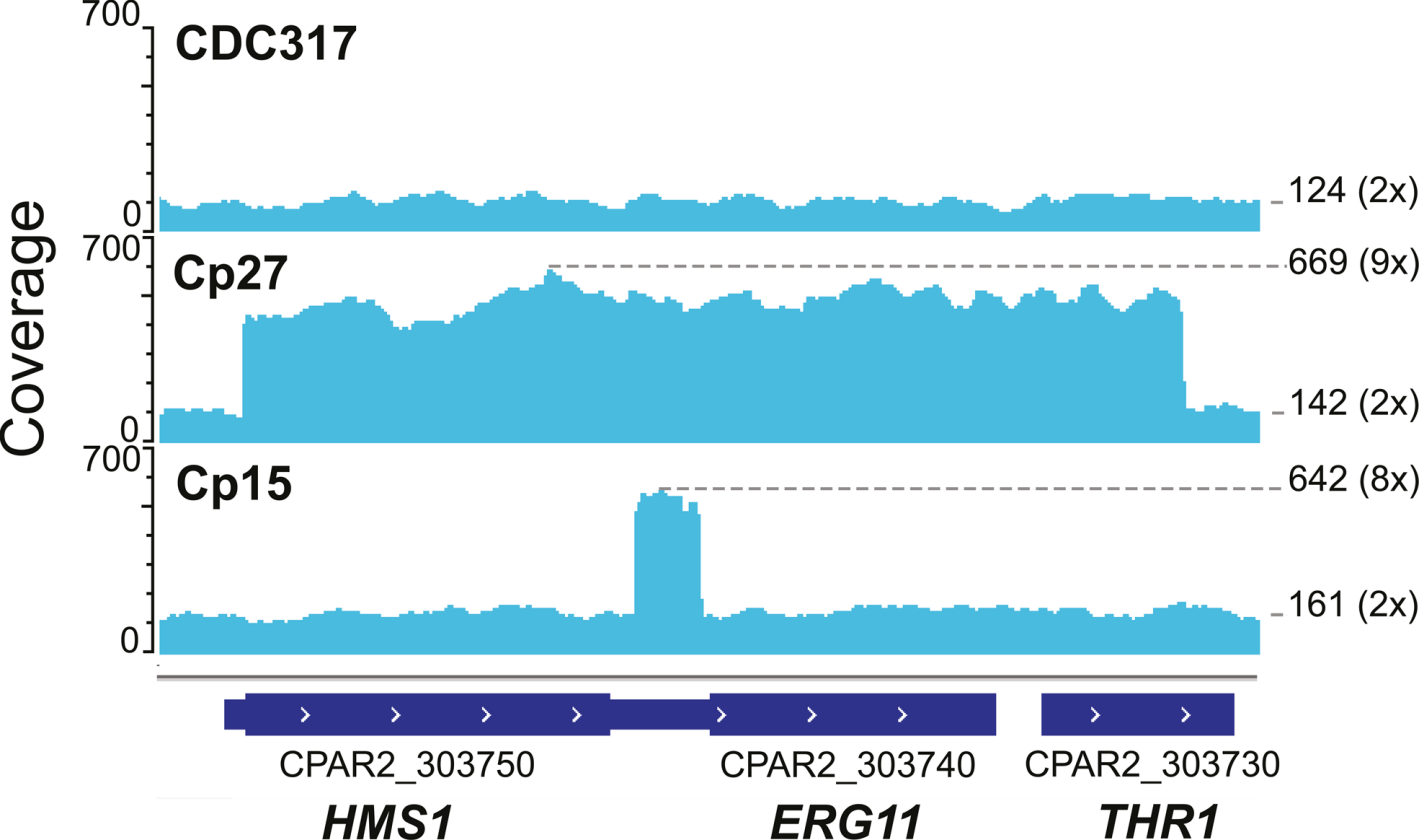
Amplification of *ERG11* in fluconazole resistant isolates *C. parapsilosis* Cp15 and Cp27. Screenshot from IGV (Integrative Genomics Viewer) showing coverage (read depth) tracks (52) for strains Cp15, Cp27, and the reference CDC317. The coverage value is marked on the right for the peak of the amplification and for the region average. The estimated copy numbers are in parentheses. Thick bars at the bottom show the CDS of genes, and thinner bars represent transcribed regions.

We found that two resistant strains, Cp15 and Cp27, have small CNVs at the *ERG11* locus on chromosome 3 (Fig. 2). In Cp27, the entire coding sequence of both *ERG11* and the upstream gene (*HMS1*), and part of the downstream gene (*THR1*), has been amplified by the CNV (1,309,908–1,315,502 bp). Here, the locus has been amplified to nine copies. Short-read mate-pair mapping supports the interpretation that this CNV is a tandem array of duplicated sequences. In Cp15, a 341 bp section of the *ERG11* promoter region is amplified to eight copies (1,312,556–1,312,896 bp). Cp15 and Cp27 are the only two isolates in this collection with expression of *ERG11* more than two times that of Cp13 (Log_2_FC values 2.38 and 2.45; Table 1). As far as we are aware, this is the first time that an amplification of an *ERG11* promoter has been observed in any *Candida* species.

### The *CDR1B* locus contains two genes, *CDR1B.1* and *CDR1B.2*, and is amplified in resistant isolates

The *CPAR2_304370* locus (the annotated *CDR1B* gene in the reference genome assembly) has increased sequence coverage compared to the genomic average in most of the isolates. Overexpression of *CDR1B* has previously been observed in fluconazole-resistant isolates and shown to directly contribute to this phenotype (40, 41, 47). In a study investigating acquired azole resistance in consecutive isolates taken from a patient undergoing fluconazole treatment, one isolate with reduced susceptibility to fluconazole had undergone amplification of the *CDR1B* locus, from 4 to ~15 copies (47). In addition, by using GWAS analysis, we identified a potential, though not statistically significant, association between fluconazole resistance and *CDR1B* (see Supplementary Text).

We used the average coverage across the ORF to estimate the copy number of *CDR1B* in each of the isolates. Whereas a majority (25/42) have a copy number in the range 4×–6×, there are several outliers ranging up to 33×, and only three isolates have the expected value for a diploid organism of 2× (Fig. 1). Several isolates that have increased expression of *CDR1B* but no corresponding *MRR1* gain-of-function mutation have increased copy number of *CDR1B*, suggesting that amplification of this locus, and activating mutations in upstream regulators, can both drive overexpression of this gene. Strikingly, two of the three isolates with two copies of the locus are susceptible to fluconazole, and no susceptible isolate has more than five copies.

Of special note is the clade containing *C. parapsilosis* FM16, CDC317, and Cp14 (Fig. 1). FM16 is susceptible to fluconazole and has only two copies of *CDR1B* (*CPAR2_304370*). CDC317 and Cp14 have MIC values of 16 and 64 µg/mL, respectively (Table 1). Although CDC317 has been previously reported to have an MIC of 4 µg/mL [susceptible dose dependent (53)], the MIC of our sequenced isolate is higher. CDC317 has only five copies of *CDR1B*. However, CDC317 is heterozygous for a Y132F mutation in *ERG11* that is not present in Cp14 (Table 1). In contrast, Cp14 has 16 copies of *CDR1B*. We propose that the related isolates Cp14 and CDC317 acquired resistance by differing mechanisms: the former by acquiring a mutation in *ERG11*, and the latter through an increased copy number of *CDR1B*. Likewise, resistant isolate Cp6 has 10 copies of *CDR1B*, and no other variant is known to be associated with azole resistance (Table 1).

The MIC values of the isolates from the Johannesburg clade range from 16–256 µg/mL (18) (Table 1). Two related isolates (Cp38 and Cp35) with MICs of 32 µg/mL have acquired a G650E substitution in *TAC1*, resulting in increased expression of *CDR1* (Table 1) (18). The combination of Y132F in *ERG11* and G650E in *TAC1* likely increases resistance compared to each variant alone (18). The copy number of *CDR1B* is highly variable in the Johannesburg isolates, ranging from 6× to 33× (Fig. 1). Whereas there is no direct correlation between the copy number of *CDR1B* and MIC in these isolates, it is notable that Cp37 has the highest MIC (256 µg/mL) and the highest number of *CDR1B* copies (~33×). In addition, although Cp37 has an estimated 33 copies of *CDR1B*, the expression of *CDR1B* is only ~3.5 times higher than susceptible strain Cp13 (Table 1). Similarly, the expression of *CDR1B* is increased by less than twofold in Cp6 (10 copies), indicating there may not be a linear relationship between a number of copies of *CDR1B* and its expression.

**Fig 3:**
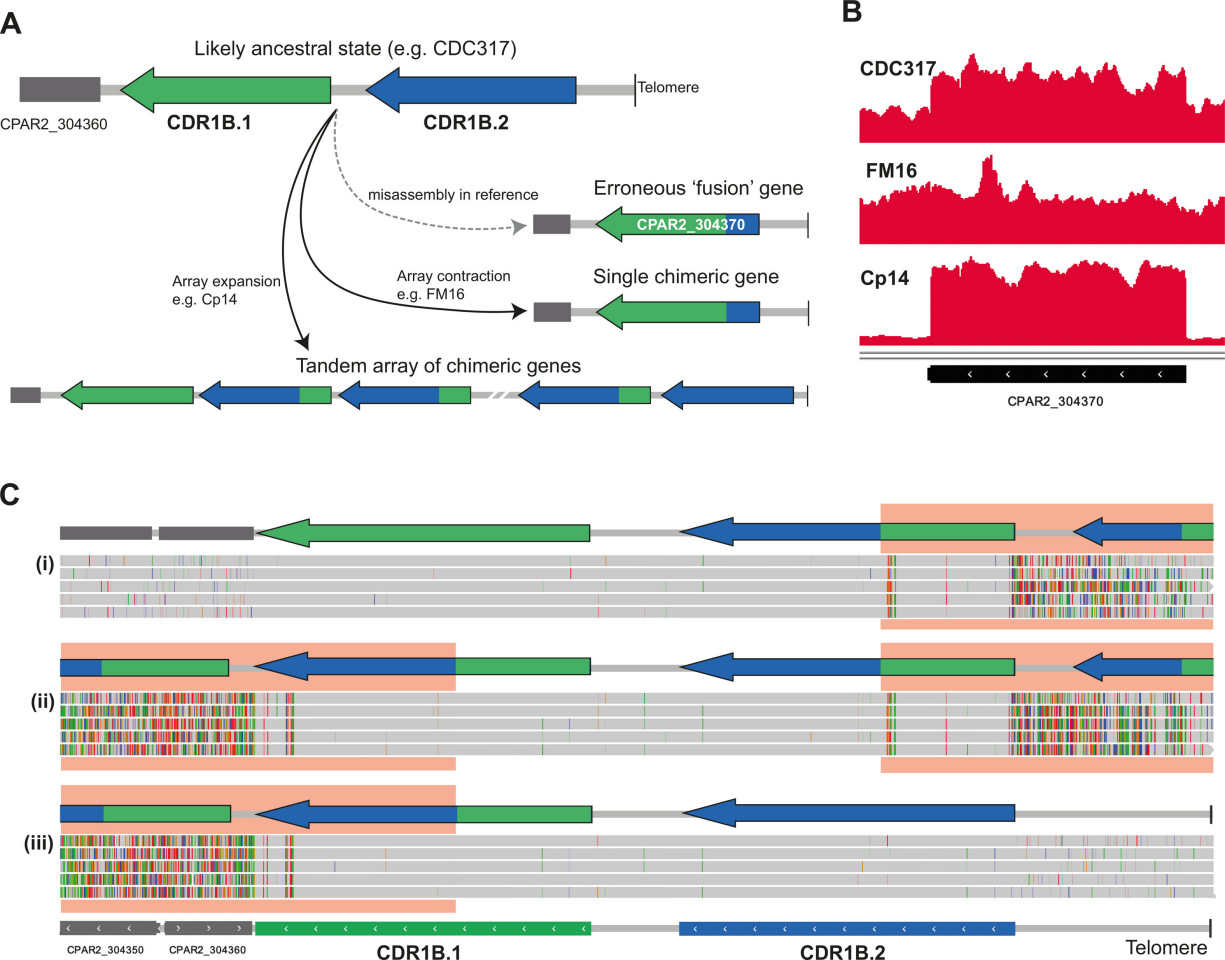
(**A**) Diagram showing the likely ancestral state of the *CDR1B* (*CPAR2_304370*) locus, with two highly similar genes *CDR1B.1* and *CDR1B.2* in tandem as occurs in the corrected genome sequence of *C. parapsilosis* CDC317 (based on MinION sequencing). The dashed gray arrow indicates how mis-assembly of this locus in the original reference genome sequence for CDC317 (54) led to an erroneous fusion between *CDR1B.1* and *CDR1B.2*, resulting in the annotation of the incorrect gene structure *CPAR2_304370*. The solid gray arrows show CNVs formed by array expansion and or contraction in other isolates, such as Cp14 and FM16. (**B**) Coverage tracks in IGV of short-read data from three isolates aligned to the original *C. parapsilosis* CDC317 assembly. Both CDC317 and Cp14 have increased coverage at the *CPAR2_304370* locus, whereas FM16 does not. (**C**) MinION reads of isolate Cp14 aligned to a long-read CDC317 assembly show evidence of a tandem array of chimeric genes bounded by parental genes. The bottom track shows the annotated long-read assembly of CDC317, containing both *CDR1B.1* and *CDR1B.2*. Each gray bar is a single read aligned to the *CDR1B* locus, where gray denotes read sequence matching the reference, and colored dashes are mismatched positions, from modified screenshots of IGV (52). Reads aligned to this locus belong to one of three groups: (i) reads that align fully to *CDR1B.1* and left flanking DNA while partially aligning to *CDR1B.2*, (ii) reads that align partially to both *CDR1B.1* and *CDR1B.2*, and (iii) reads that align fully to *CDR1B.2* and right flanking DNA while partially aligning to *CDR1B.1*. A schematic showing the genic content for each read group is shown above for clarity. Red boxes highlight regions where the read sequence does not match the reference sequence. Reads from groups (i) and (iii) contain one parent gene and one or more copies of the tandem chimera. Reads from the group (ii) contain multiple copies of the tandem chimera but neither of the parent genes.

Long-read (Oxford Nanopore) sequencing of CDC317 revealed that the *CPAR2_304370* (*CDR1B*) gene annotated in the Sanger-sequencing reference genome assembly of this strain was in fact erroneously assembled by fusing together two highly similar tandem genes, which we now call *CDR1B.1* and *CDR1B.2* (Fig. 3A). As a result, the intergenic space between these two genes, and parts of the genes themselves, is not present in the original reference assembly. It is likely that the presence of *CDR1B.1* and *CDR1B.2* is the ancestral (and most common) state of the locus in *C. parapsilosis* (Fig. 3A), and the four copies of *CPAR2_304370* indicated in many of the isolates by coverage analysis relative to the reference genome assembly in fact represent diploids with two copies of both *CDR1B.1* and *CDR1B.2* (Fig. 1). We used long sequencing reads, alongside short reads, to generate a highly accurate, complete chromosome assembly of CDC317. This assembly confirmed that CDC317 has two copies each of *CDR1B.1* and *CDR1B.2*, so the previously estimated copy number of 5× *CPAR2_304370* (Fig. 1) was likely inflated by short reads mismapping from related genes. Using the new accurate assembly of CDC317, we found that *CDR1B.1* and *CDR1B.2* are 98.69% identical at the nucleotide level, and the intergenic regions upstream of both genes are 46.10% identical. The two genes differ by only 23 amino acids (out of 1,498) when translated.

Significantly, short reads from isolate FM16 (estimated to have two copies of *CPAR2_304370*) map to the original *C. parapsilosis* CDC317 reference genome without an increase in coverage or misaligned read pairs (Fig. 3B). This is evidence of an array contraction of the two genes in FM16 that results in a single chimeric *CDR1B.2/CDR1B.1* gene, biologically mirroring the misassembly in the original reference. Short-read alignments of Cp36 and Cp5 suggest that similar array contractions occurred in these isolates.

Long-read sequencing of Cp14 revealed that the extra copies predicted by coverage analysis (16× *CPAR2_304370*) are the result of a tandem array of identical chimeric *CDR1B.1/CDR1B.2* genes, bounded upstream by non-chimeric *CDR1B.2* and downstream by non-chimeric *CDR1B.1* (Fig. 3A). These chimeric genes inherited their 5’ region from *CDR1B.1* and their 3’ region from *CDR1B.2*. In this manner, they are opposite to the chimeric gene in FM16. None of the Cp14 long sequencing reads reached across the entire tandem array, so the exact copy number of the chimeric genes could not be determined. However, by aligning the reads to the long-read assembly of CDC317, we found reads that contain the beginning, middle, and end of the tandem array (Fig. 3C).

### Families of *CDR* orthologs and paralogs in *Candida* spp

**Fig 4:**
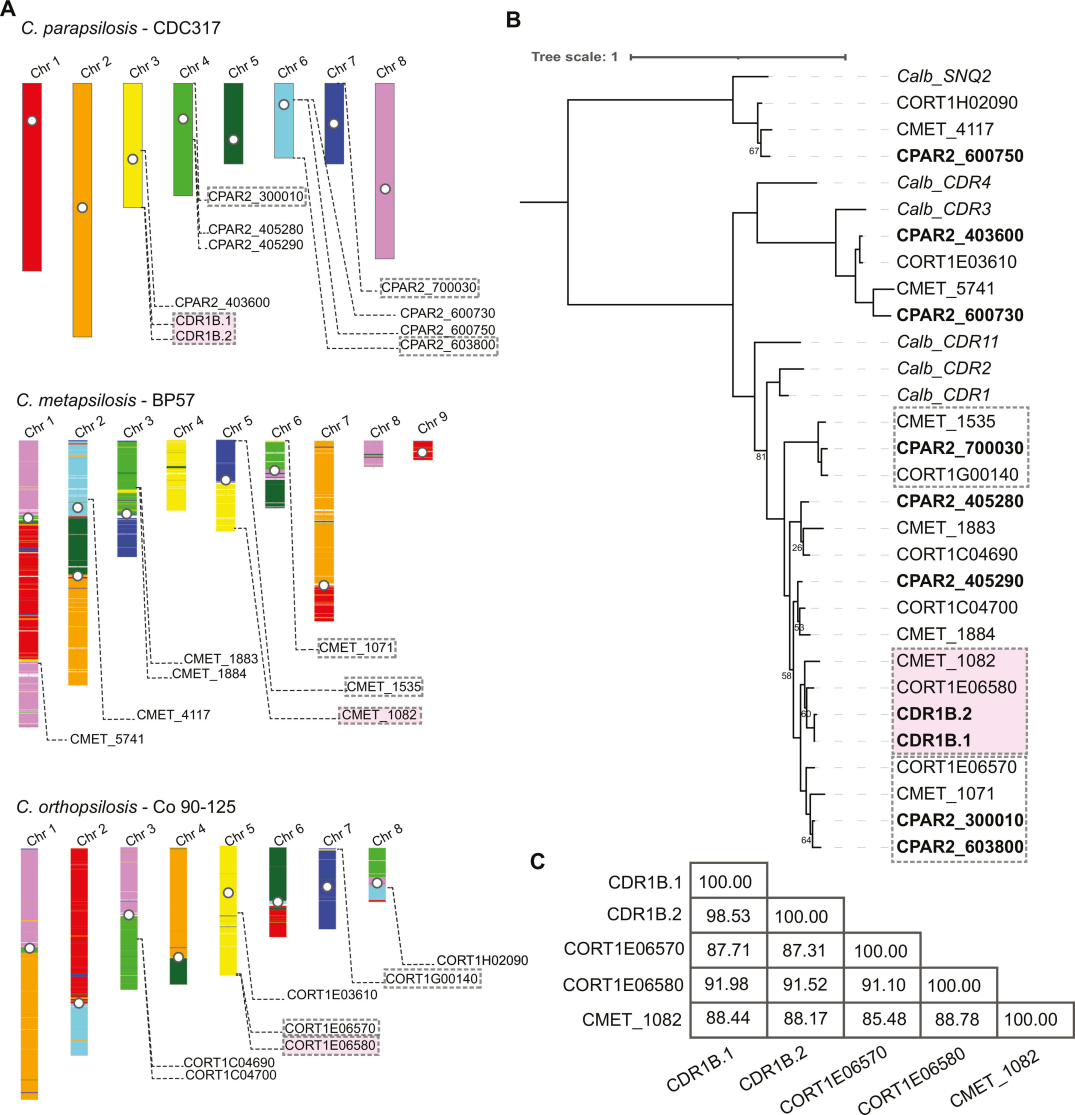
(**A**) Schematic of synteny shared between members of the *C. parapsilosis* species complex. The *C. parapsilosis* CDC317 long-read genome assembled in this study was aligned to the genomes of *C. metapsilosis* (GCA_017655625.1) (55) and *C. orthopsilosis* (PRJNA767198) using BLASTN. These genomes were then colored according to the *C. parapsilosis* chromosome aligned to each region. Previously identified centromeres are marked with a white circle (56). The locations of *CDR* genes are shown for each species. (**B**) Phylogenetic tree of CDR protein sequences from *C. parapsilosis*, *C. orthopsilosis*, *C. metapsilosis*, and *C. albicans*. Pink backgrounds denote genes most closely related to *CDR1B.1* and *CDR1B.2*. Three clades of genes located near telomeres are marked with boxes. Protein sequences were aligned using the Clustal Omega method in Seaview (57). The tree was constructed with the LG model within Seaview, using 100 bootstrap replicates. Bootstrap values > 85 have been omitted for clarity. (**C**) Percent identity matrix for the five *CDR* genes syntenic to the *CDR1B* locus in the *C. parapsilosis* species complex.

*CDR1B.1* and *CDR1B.2* are two of nine *CDR* genes in *C. parapsilosis* (Fig. 4). Strikingly, most (5/9) of these genes are located in telomeric regions (Fig. 4A). Many of the *CDR* genes in *C. parapsilosis*, including all five telomeric ones, have direct orthologs in *C. metapsilosis* and *C. orthopsilosis*, but they are more distantly related to *C. albicans CDR* genes (Fig. 4B). The *CDR* orthologs in *C. metapsilosis* and *C. orthopsilosis* share synteny of neighboring genes when compared to the gene order of *C. parapsilosis*. The telomeric *CDR* genes are likely to have originated after *C. parapsilosis* diverged from the *C. albicans* lineage but before the separation of *C. parapsilosis* from *C. orthopsilosis* and *C. metapsilosis*. In addition, a recent gene duplication in *C. parapsilosis* produced the gene pair *CPAR2_300010/CPAR2_603800* which is duplicated only in this species, while the *CDR1B.1/CDR1B.2* gene pair has a more complicated history.

*C. metapsilosis* has only a single gene (*CMET_1082*) at the *CDR1B* locus, which is equally related to the two *C. parapsilosis CDR1B* genes (Fig. 4B and C; genes highlighted in pink). Long-read sequences of the reference *C. metapsilosis* strain BP57, recently assembled by Mixão et al. (55), indicate that this is not due to misassembly. There are two *CDR* genes present at the *CDR1B* locus in *C. orthopsilosis*, but interestingly, only one of them (*CORT1E06580*) falls phylogenetically into the same group as *C. parapsilosis CDR1B.1* and *CDR1B.2* and *C. metapsilosis CMET_1082*, while its neighbor (*CORT1E06570*) falls into an adjacent group with *CMET_1071* and two *C. parapsilosis* genes *CPAR2_300010* and *CPAR2_603800* (Fig. 4B). *CORT1E06580* is ~91% identical to *CDR1B.1* and *CDR1B.2*, while *CORT1E06570* is ~87% identical (Fig. 4C). Notably, the *CDR1B* locus, *CPAR2_300010* and *CPAR2_603800* are all telomeric on different chromosomes of *C. parapsilosis*, so we suggest that there may have been some *CDR* gene exchange and/or homogenization among telomeric regions within these species.

We did not observe CNVs affecting any of the other *CDR* genes in *C. parapsilosis*, nor in 36 *C*. *orthopsilosis* or 30 *C*. *metapsilosis* isolates that we analyzed. The *CDR1B* locus of *C. parapsilosis* is unique among the *CDR* genes of these three species in having two highly similar genes in tandem, which provides a template for amplification of the locus to occur readily.

## DISCUSSION

Using a genome-wide approach, we identified two CNVs (*ERG11* and *CDR1B.1*/*CDR1B.2*) that are associated with fluconazole resistance in *C. parapsilosis*. CNVs are a method of gene duplication by which an organism can transiently adapt to its environment (58, 59). Environmental changes, such as the introduction of an antifungal drug, can select for specific genes to be duplicated, and thereby overexpressed (50, 60). After the drug is removed, the CNV can be lost by selective pressure to maintain a compact genome size (60).

Overexpression of *ERG11* by increasing copy number has been observed in *C. albicans* (48, 49). However, in *C. albicans*, the gene was amplified along with *TAC1* by means of a partial aneuploidy of chromosome 5 leading to the formation of an isochromosome, i(5L). The i(5L) isochromosome, which typically results in a single extra copy of the chromosomal region, has been identified in multiple clinical isolates, where it has a modest but measurable impact on fluconazole resistance in different genetic backgrounds (48, 50, 61). Genomic expansion of *ERG11* in combination with hotspot mutations is also associated with azole resistance of *C. tropicalis* (62). In *C. auris*, a large survey of 304 isolates identified a CNV including *ERG11* in 18 isolates (most from a single clade), which was associated with fluconazole resistance (63). Recently, a laboratory-directed evolution experiment also showed that reduced azole susceptibility is associated with large segmental duplications containing *ERG11* in *C. auris*. One evolved strain had a 191 kb long CNV with 75 protein-encoding genes including *ERG11* amplified, while another had a 161 kb long CNV containing 67 protein-encoding genes including *ERG11* (64). Despite an extra copy of chromosome 3, which contains both *ERG11* and *TAC1* in *C. parapsilosis*, the isolate Cp3 in this study is susceptible to fluconazole. It is possible that the increased level of *ERG11* and *CDR1B* (log_2_FC ~ 0.8) is not sufficient to drive resistance.

A very recent analysis identified amplifications of *ERG11* in 21 azole-resistant isolates of *C. parapsilosis* (65). The amplifications ranged from partial aneuploidy of chromosome 3, similar to *C. albicans* (48, 50), to smaller amplifications of 2.3–12.1 kb (65). These are similar to the amplifications that we observe in strain Cp27, where *ERG11* and its neighboring gene are amplified (5.6 kb). The CNV in strain Cp15 is distinctly different; only the *ERG11* promoter region is amplified. Importantly, both Cp27 and Cp15 isolates also have increased *ERG11* expression (log_2_FC ~ 2.4; Table 1), strongly suggesting these duplications directly impact the expression of this gene and fluconazole resistance. We have previously observed a similar localized gene amplification in *C. parapsilosis* in the gene *RTA3* (44). Several different CNVs, spanning either the whole gene or just the promoter region, led to overexpression of *RTA3* associated with increased resistance to the antimicrobial drug miltefosine. Promoter amplifications may, therefore, be a previously underexplored mechanism of drug resistance in *Candida* species.

We attempted to use GWAS analysis to identify variants associated with fluconazole resistance. However, no significant associations were found (Supplementary Text). Although disappointing, it is within expectation because the sample size (*n* = 42) is very low for a study of this kind, and there was a large imbalance between the number of resistant (*n* = 35) and susceptible (*n* = 7) isolates. PowerBacGWAS, a tool used to find required sample sizes for GWAS in bacteria, estimates that in a best-case scenario where minor allele frequency is high and effect size is large, analysis of 500 isolates would be required to identify a single significant SNP in *Mycobacterium tuberculosis* (66). *M. tuberculosis* is strictly clonal with no evidence of gene transfer (67), making it a useful proxy for other clonal organisms such as *C. parapsilosis*. This issue is further compounded in our study by the presence of groups of highly related strains from persistent clinical lineages, which reduces the effective sample size. Despite the lack of statistical power, the GWAS results pointed to *CDR1B* amplification as a possible mechanism of fluconazole resistance. Further studies on the use of GWAS in *C. parapsilosis* with higher sample sizes are necessary to determine whether GWAS as a tool is appropriate for this organism.

Our analysis of the *CDR* family in the *C. parapsilosis* species complex has led to some interesting insights. We found that most of the *CDR* genes in the *C. parapsilosis* species complex have arisen from gene duplication events after the lineage diverged from *C. albicans*. The *CDR* gene content varies between *C. parapsilosis* and its two sister species. Many *CDR* copies are located at telomeres, and there is evidence of the exchange of duplicated genes between telomeres. The *CDR1B* locus is particularly variable between the three sister species; there is a single gene in *C. metapsilosis*, two distinct genes in *C. orthopsilosis*, and two highly similar genes in *C. parapsilosis*. It is possible that *C. orthopsilosis* represents the ancestral state, with one of the genes lost in the *C. metapsilosis* lineage and one gene overwritten in *C. parapsilosis* by gene conversion from its neighbor.

Previous analysis identified several potential azole resistance mechanisms in some of the strains described here (18, 40, 41). This includes the Y132F mutation in *ERG11* (Table 1) (18). We find that this variant is present in all the closely related isolates from Johannesburg, supporting our inference that these represent a single lineage. However, the range of MICs observed in these isolates cannot fully be explained by the presence of Y132F (Table 1). Two isolates (Cp35 and Cp38) collected in 2008 have also acquired variants in Tac1, with an associated rise in expression of *CDR1* (log_2_FC 0.4–0.7; Table 1) (18). Our analysis suggests that in other isolates (e.g., Cp37, also collected in 2008), increased resistance is associated with, and may be caused by, amplification of the *CDR1B* locus (up to ~33 copies).

The presence of *ERG11* Y132F is increasingly associated with infection outbreaks (19–22, 24, 25). However, outbreaks can also be caused by strains without Y132F such as the Bloemfontein isolates in this study. For example, in a study investigating 60 *C*. *parapsilosis* strains involved in a large outbreak in a Brazilian ICU, only ~36% of isolates resistant to fluconazole had an *ERG11* mutation (68). Another outbreak among patients undergoing allo-hematopoietic cell transplant treatment was associated with isolates without the Y132F mutation (69).

All strains from the Bloemfontein clade have increased expression of *MDR1B* (log_2_FC 1.8–5.28, Table 1). Although the isolates are closely related, there is some variability in their *MRR1* alleles. Some isolates contain an A854V-activating mutation in *MRR1*, which is known to result in overexpression of *MDR1B* (41). Seven are heterozygous for the A854V mutation in *MRR1*, one is homozygous for the mutation, and six do not have the mutation. The earliest cultured strains (Cp1 and Cp2 isolated in 2001) are both heterozygous for the mutation, whereas some strains isolated later (e.g., Cp17 in 2003) are lacking the mutation entirely (Table 1). This indicates that there may be sub-populations of related strains existing concurrently in the clinic or the local population which are variable at *MRR1*. Multiple strains without Mrr1 A854V have increased expression of *MDR1B* (Table 1), showing that there may be additional unidentified factors contributing to *MDR1B* expression in this clade.

Eleven of the resistant isolates in Table 1 contain none of the common variants shown experimentally to be associated with azole resistance. Our work identifies relevant CNVs in four of these. *ERG11* is amplified in two strains, Cp27 and Cp15, that are not members of the South African clades. Strains Cp14 and Cp6, which are also not in these clades, both have >10 copies of *CDR1B* and no other identified resistance mechanism. The variation in the copy number of *CDR1B* across the isolates suggests that *CDR1B* amplification may be a common mechanism of azole resistance in *C. parapsilosis*. However, even among strains with an increased copy number of *CDR1B*, there is a range of MIC values, and higher copy numbers do not necessarily correlate with higher MIC. It is likely that there are additional, as of yet unidentified, mechanisms that contribute to resistance in these strains. It is also notable that we have not identified any potential causative variants in seven isolates (Cp8, Cp11, Cp16, Cp17,Cp18, Cp19, and Cp32), suggesting that other drug resistance mechanisms remain to be identified.

Of the multiple *CDR* genes in multiple *Candida* species, we observed CNVs at only the *C. parapsilosis CDR1B* locus. We propose that the existence of two nearly identical genes in tandem makes CNVs at this locus more likely to occur. In this way, *C. parapsilosis* may be primed to generate extra copies of *CDR1B*, and therefore be predisposed to develop fluconazole resistance. Amplification likely occurs during infection as described by Branco et al. (47), where a strain of *C. parapsilosis* in a patient treated with fluconazole acquired a CNV amplifying *CDR1B* that was associated with reduced fluconazole susceptibility.

## MATERIALS AND METHODS

### RNA sequencing

*C. parapsilosis* isolates were maintained at −80°C in 40% glycerol stocks. Isolates were grown in yeast peptone dextrose (YPD) liquid media overnight and plated onto Sabouraud-Dextrose (BD companies) agarose plates in biological triplicate for 24 h growth at 30°C. Sterile loops were used to inoculate 20 mL RPMI with MOPS and 2% glucose to OD600 = 0.1. Cultures were incubated at 35°C with 110 rpm shaking for 8 h, after which the cells were centrifuged at 4,000 rpm for 5 min. Supernatants were removed, and pellets were stored at −80°C for a minimum of 24 h. RNA isolation was performed via RiboPureTM Yeast (Invitrogen) kits per the manufacturer’s instructions. RNA-sequencing was performed using Illumina NextSeq for stranded mRNA (Hartwell Center, St. Jude Children’s Research Hospital). Libraries were prepared with paired-end adapters using Illumina chemistries per the manufacturer’s instructions, with read lengths of approximately 150 bp with at least 50 million raw reads per sample (Bioproject 14022043). RNA-sequencing was analyzed using CLC Genomics Workbench version 20.0 (QIAGEN), and reads were trimmed using default settings for failed reads and adaptor sequences and then subsequently mapped to the *C. parapsilosis* genome (GenBank accession: GCA_000182765.2) with paired reads counted as one and expression values set to RPKM. Principal component analysis was utilized for the initial assessment of biological replicate clusters. Whole transcriptome differential gene expression analysis was performed with the prescribed algorithm of CLC Genomics Workbench version 20.0. Mismatch, insertion, and deletion costs were set to default parameters, and a Wald test was used to compare all isolates against the fluconazole-susceptible isolate Cp13. Fold changes for CPAR2_304370, CPAR2_405290, CPAR2_301760, CPAR2_603010, and CPAR2_303740 were identified for all isolates and are reported in Table 1.

PCA was performed on the 36 sequenced strains using normalized read counts (RPKM) for all genes, using the scikit-learn module in Python.

### Whole-genome sequencing

Genomic DNA was isolated from overnight YPD liquid media cultures utilizing a Triton SDS and phenol-chloroform method previously described by Amberg et al. (70). DNA concentrations were quantified using both the Qubit Fluorometer and Nanodrop spectrophotometer using the manufacturer’s protocols. Whole-genome libraries were prepared and sequenced on the NovaSeq600 platform (150 bp, paired-end reads) by the University of Maryland School of Medicine Institute for Genomic Sciences.

### Fluconazole susceptibility testing

Inoculums of YPD liquid media were prepared from original stocks and stored at −80°C in 40% glycerol. Inoculates were grown at 30°C with 220 rpm shaking overnight and subsequently plated onto Sabouraud dextrose agar for overnight incubation at 35°C. MICs were determined in RPMI1640 (Roswell Park Memorial Institute) supplemented with MOPS [3-(N-morpholino) propanesulfonic acid] buffer and 2% glucose, pH 7.0, liquid media following CLSI M27-A4 methods for broth microdilution. Fluconazole (Sigma Aldrich) drug stocks were prepared in dimethyl sulfoxide at 100 × the maximum plate concentration (256 mg/mL for resistant isolates and 16 mg/mL for susceptible isolates). MICs were determined visually as the concentration achieving 50% growth inhibition at 24 hours, and the modal value of biological triplicate measurements was considered the MIC reporting.

### MinION sequencing

Samples were grown overnight in 50 mL of YPD broth at 30°C. Genomic DNA was extracted from 1.5 mL of liquid cultures saturated to 10 A600 units per millilitre using the Yeast Masterpure DNA purification kit (MPY80010) following the manufacturer’s instructions.

Genomic DNA (1 µg) from each sample was sequenced with MinION technology using the native barcoding kits (SQK-NBD-24 and SQK-NBD114-24) from Oxford Nanopore Technologies (ONTs), following the manufacturer’s instructions. Library kit SQK-NBD112-24 was used for sequencing CDC317 on an R9.4.1 chemistry flowcell (FLO-MIN106D). Library kit SQK-NBD114-24 was used for sequencing Cp14 on an R10.4.1 chemistry flowcell (FLO-MIN114). Sequencing of both strains was performed on a MinION MK1C device with MinKNOW (ONT) versions 21.11.6 and 22.10.7 for CDC317 and Cp14, respectively. Both runs were set to the default fast configurations. Basecalling and demultiplexing were run within MinKNOW during sequencing. This generated 638,855 and 237,020 raw reads for CDC317 and Cp14, respectively.

### Sequence analysis

The Illumina reads for all 42 *C*. *parapsilosis* samples were downsampled to ~100× coverage using the Picard version 2.21.2 DownsampleSam on unmapped SAM files. These files were converted to FASTQ format using Picard SamToFastq and aligned to the *C. parapsilosis* CDC317 reference genome using bwa mem version 0.7.17 (71). GATK version 4.1.4.1 was used to mark duplicate reads and reorder the mapped BAM files with the tools MarkDuplicates and ReorderSam, respectively (72). Variants were called on individual samples using GATK HaplotypeCaller with the “-ERC GVCF” tag. The GVCF outputs were combined into a multi-sample VCF with GATK CombineGVCFs and then genotyped using GATK GenotypeGVCFs. SNP variants were hard filtered using GATK VariantFiltration with following parameters: QD < 2.0, QUAL < 30.0, SOR > 3.0, FS > 60.0, MQ < 40.0, MQRankSum < −12.5, ReadPosRankSum < −8.0, and DP < 10. Indels were similarly filtered using parameters: QD < 2.0, QUAL < 30.0, FS > 200.0, and ReadPosRankSum < −20.0. All variants were filtered using GATK SelectVariants to remove multi-allelic sites and sites that contained >10% “no-call” genotypes. The two sets of variants were combined for GWAS analysis, and a file containing only SNPs was used for phylogenetic analysis. MinION reads for isolate CDC317 were filtered using NanoFilt to exclude reads with quality <12 and length <10,000 (73). The filtered reads were assembled using Canu version 2.2 (74). Errors in this assembly were removed by incorporating Illumina read data using NextPolish version 1.4.0 (75). The assembly is available under accession PRJNA1031570. Reads for *C. orthopsilosis* were obtained from Schröder et al. (76), Pryszcz et al. (77), Bergin et al. (44), and Zhai et al. (69), and reads for *C. metapsilosis* were obtained from Zhai et al. (69) and O’Brien et al. (78) (Table S3).

### Phylogeny

A FASTA alignment of all sites containing an SNP in at least one isolate was created from the multi-sample VCF file using a custom script (https://github.com/CMOTsean/HetSiteRando). Heterozygous variants were randomly assigned to either allele on a per-site basis. An SNP tree was constructed with the alignment file using RAxML version v8.2.12 with the GTRGAMMA model of nucleotide substitution and 1,000 bootstrap replicants (45).

### Estimating repeat copy number and structure

To calculate the estimated copy number for all *C. parapsilosis* genes, the short-read alignments (as described above) against the CDC317 reference genome were used. The average coverage across the ORF in each isolate was found using BEDTools coverage version 2.29.2 and then divided by half the modal genome coverage (BEDTools genomecov) for that isolate (79). To generate coverage track plots used for detecting aneuploidy, the average coverage was found for 1 kb windows across the genome. Again, the values were divided by half the modal genome coverage, and then log transformed and plotted using the matplotlib library in Python.

To confirm copy number estimates and investigate the structure of the repeat, the nucleotide sequence of *CDR1B.1* was searched against MinION reads from strains CDC317 and Cp14 using BLASTN. Additionally, the Cp14 reads were aligned to the MinION CDC317 assembly using GraphMap version 0.3.0 (80).

### *C. parapsilosis* species complex comparison

To create the synteny maps between *C. parapsilosis* and its sister species, the *C. parapsilosis* CDC317 reference genome was aligned against the *C. metapsilosis* BP57 reference genome (GCA_017655625.1) (55) and the *C. orthopsilosis* SY36 long-read assembly (PRJNA767198) using BLASTN. Hits from each query chromosome were assigned a color and then plotted.

To construct the tree of CDR protein sequences, the sequences for *C. albicans*, *C. parapsilosis*, and *C. metapsilosis* were taken from the Candida Gene Order Browser(CGOB, http://cgob3.ucd.ie/), with the exception of *CMET_1535*, which came from the *C. metapsilosis* BP57 assembly.

## Data Availability

DNA sequence assembly and raw data are available under accession PRJNA1031570, and RNA sequencing is available at BioProject PRJNA1053316.

## SUPPLEMENTAL MATERIAL

Text S1 aac.01619-23-s0001.docx GWAS description.

Fig. S1 aac.01619-23-s0002.pdf Maximum-likelihood tree of 207 *Candida parapsilosis* isolates.

Fig. S2 aac.01619-23-s0003.pdf Principal component analysis of RNA-seq data.

Fig. S3 aac.01619-23-s0004.pdf Genome-wide coverage analysis of selected isolates.

Table S1 aac.01619-23-s0005.xlsx RNA-seq data for 37 strains.

Table S2 aac.01619-23-s0006.xlsx Estimated copy number for all *C. parapsilosis* genes for all 42 strains.

Table S3 aac.01619-23-s0007.docx Source of sequencing data for isolates.

Table S4 aac.01619-23-s0008.xlsx SNPs included in GWAS analysis.

ASM does not own the copyrights to Supplemental Material that may be linked to, or accessed through, an article. The authors have granted ASM a non-exclusive, world-wide license to publish the Supplemental Material files. Please contact the corresponding author directly for reuse.
