# Supplementary material for: Analysis of clinical Candida parapsilosis isolates reveals copy number variation in key fluconazole resistance genes
Source: Antimicrob Agents Chemother. 2024 May 7;68(6):e01619-23. doi: 10.1128/aac.01619-23 (PMC11620501; doi:10.1128/aac.01619-23)
Supplement: Text S1 — GWAS description. [file aac.01619-23-s0001.docx]

**Genome Wide Association Study**

We performed a Genome Wide Association Study (GWAS) using all 42 isolates to identify potential variants associated with fluconazole resistance that had not been found in earlier studies. The GWAS was carried out using GEMMA (Genome-wide Efficient Mixed Model Association) (1), which calculates and incorporates relatedness data between isolates in order to minimise the confounding effect of population structure on association scores. Because the MIC assays for fluconazole were measured in two different laboratories (Main Text, Table 1), the phenotypic data was converted to a binary score of either resistant or susceptible to reduce possible bias, with MIC ≤ 2 μg/ml classed as susceptible and MIC ≥ 8 μg/ml classed as resistant, in line with CLSI (2) and EUCAST (3) guidelines. Genotypes were also converted into binary presence/absence of non-reference alleles, with both heterozygous and homozygous variants treated as present. In addition, only variants that were predicted to affect protein function by SIFT (4) were included in the analysis to narrow the search. In total, 7462 variants were used as input to GEMMA. The GWAS analysis did not identify any significant associations below the Bonferroni-corrected p-value threshold of 6.7 x 10^-6^ (Table S4). However, several of the variants with the lowest p-value scores were in *CPAR2_405290* (*CDR1*) and *CPAR2_304370* (*CDR1B*), members of the ABC family of putative drug transporters. Investigating the alignments leading to these calls showed that the *CPAR2_405290* variants are likely a result of mis-mapping from a similar gene in the genome, so we did not investigate *CPAR2_405290* further. We investigated the *CDR1B* locus in more depth as described in the main text.

**Methods**

A binary phenotype matrix where all samples were scored as either resistant or susceptible was used as input to the GWAS. Variants entering the GWAS were filtered to only those likely to affect protein function, as annotated by SIFT4G (74). PLINK version 1.90b6.21 was used to reformat input data into BED, BIM, and FAM files for the GWAS (5). GEMMA version 0.98.5 was used to create a relatedness matrix between all strains, and then to conduct the GWAS itself using parameters ‘-hwe 0.0 -maf 0.0’ (1).

**References**

1. Zhou X, Stephens M. 2014. Efficient multivariate linear mixed model algorithms for genome-wide association studies. Nature methods 11:407-409.

2. Humphries RM, Ambler J, Mitchell SL, Castanheira M, Dingle T, Hindler JA, Koeth L, Sei K. 2018. CLSI methods development and standardization working group best practices for evaluation of antimicrobial susceptibility tests. Journal of clinical microbiology 56:10.1128/jcm. 01934-17.

3. Arendrup MC, Friberg N, Mares M, Kahlmeter G, Meletiadis J, Guinea J, Andersen C, Arikan-Akdagli S, Barchiesi F, Chryssanthou E. 2020. How to interpret MICs of antifungal compounds according to the revised clinical breakpoints v. 10.0 European committee on antimicrobial susceptibility testing (EUCAST). Clinical Microbiology and Infection 26:1464-1472.

4. Vaser R, Adusumalli S, Leng SN, Sikic M, Ng PC. 2016. SIFT missense predictions for genomes. Nature protocols 11:1-9.

5. Purcell S, Neale B, Todd-Brown K, Thomas L, Ferreira MA, Bender D, Maller J, Sklar P, De Bakker PI, Daly MJ. 2007. PLINK: a tool set for whole-genome association and population-based linkage analyses. The American journal of human genetics 81:559-575.
