# Supplementary material for: Analysis of clinical Candida parapsilosis isolates reveals copy number variation in key fluconazole resistance genes
Source: Antimicrob Agents Chemother. 2024 May 7;68(6):e01619-23. doi: 10.1128/aac.01619-23 (PMC11620501; doi:10.1128/aac.01619-23)
Supplement: Fig. S1 — Maximum-likelihood tree of 207 Candida parapsilosis isolates. [file aac.01619-23-s0002.pdf]

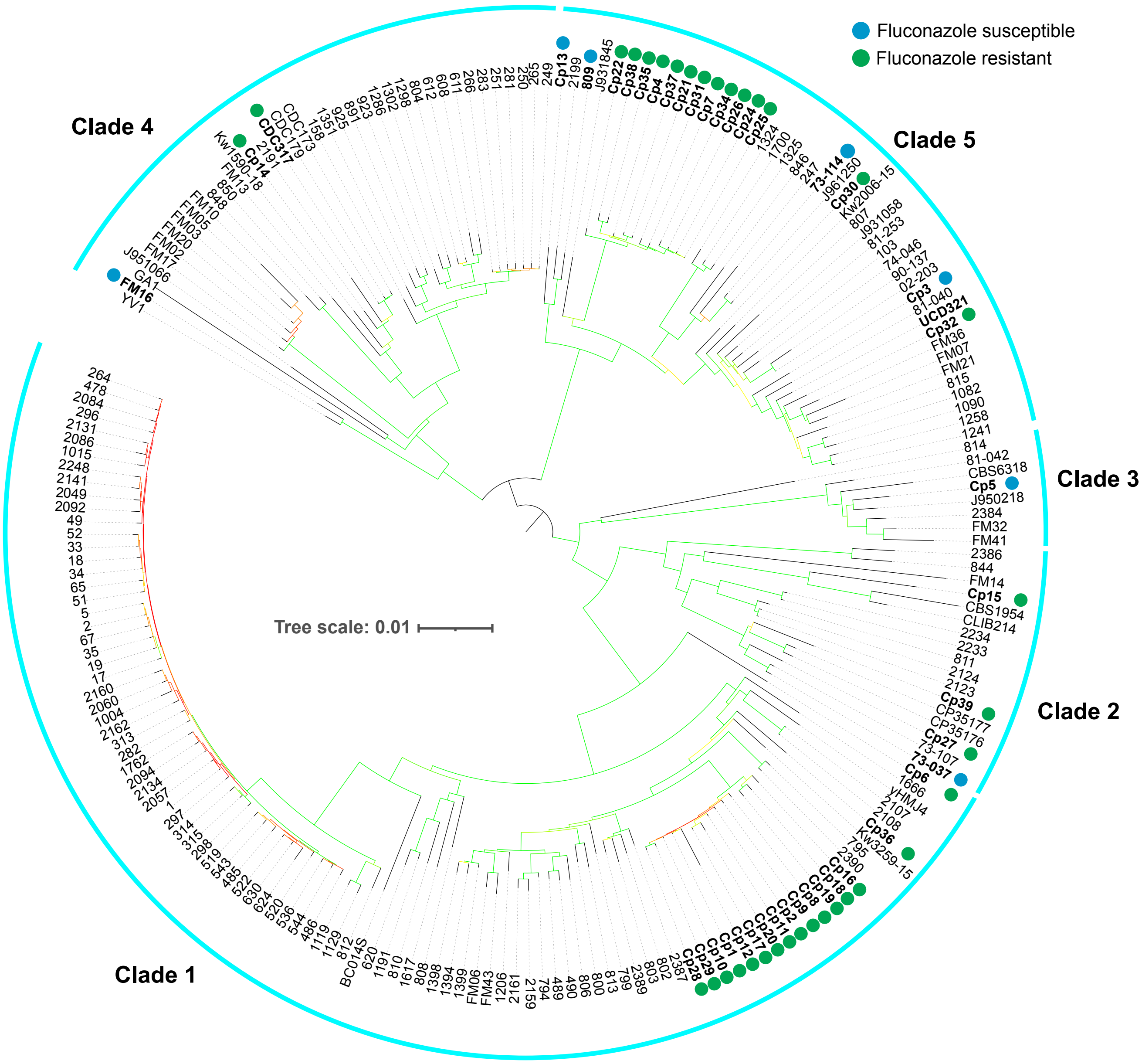

Figure S1.

Maximum-likelihood tree of 207 Candida parapsilosis isolates constructed as in Figure 1. Additional isolates, and clade designations were taken from (44). Susceptible isolates in this paper are marked with a blue circle, and resistant isolates are marked with a green circle. Coloured branches indicate bootstrap values after 1000 iterations of bootstrap sampling for that branch ranging from 0 (red) to 100 (green).
