# Supplementary material for: Analysis of clinical Candida parapsilosis isolates reveals copy number variation in key fluconazole resistance genes
Source: Antimicrob Agents Chemother. 2024 May 7;68(6):e01619-23. doi: 10.1128/aac.01619-23 (PMC11620501; doi:10.1128/aac.01619-23)
Supplement: Fig. S2 — Principal component analysis of RNA-seq data. [file aac.01619-23-s0003.pdf]

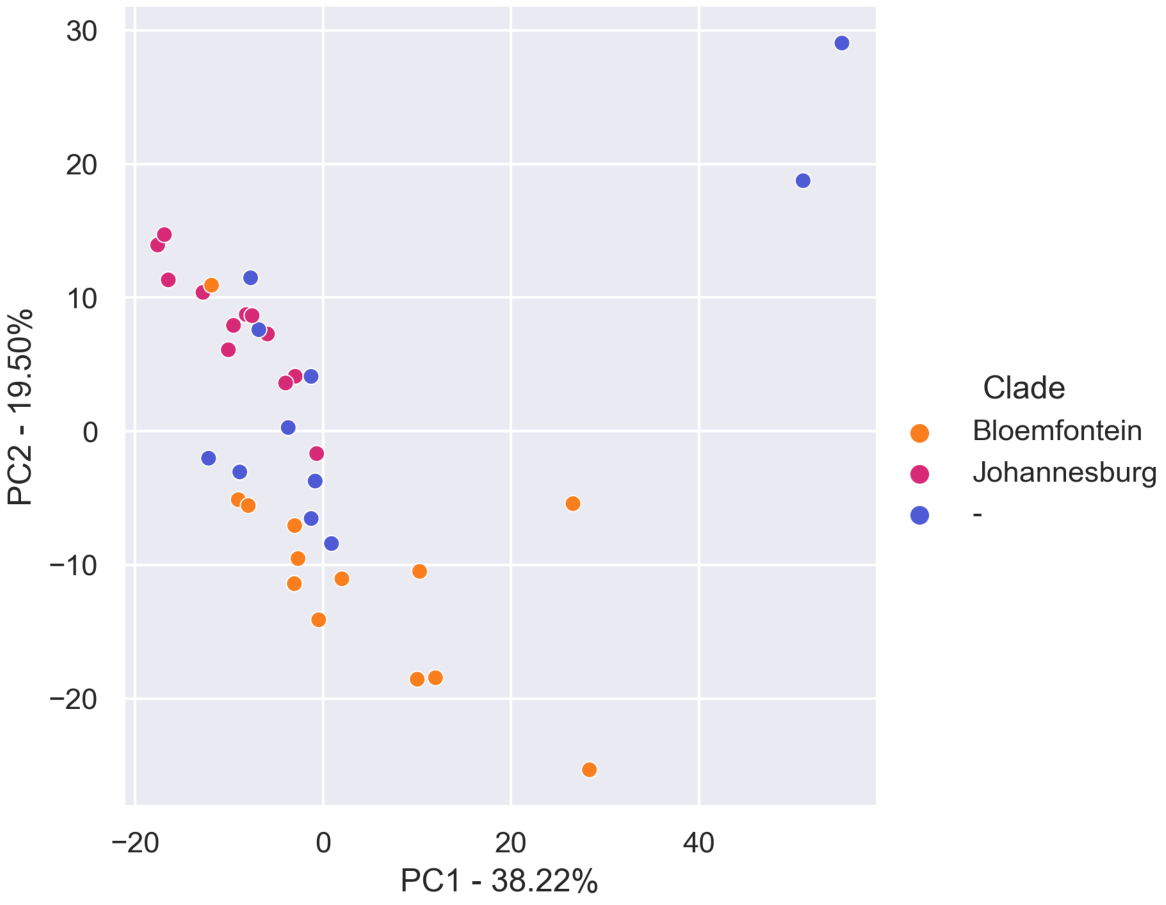

Figure S2.

Principal component analysis plot created with the scikit-learn module in Python using normalized read counts of the RNAseq data. PC1 explains 38.22% of the variance of the data and PC2 explains 19.5%.

!
