# Supplementary material for: Analysis of clinical Candida parapsilosis isolates reveals copy number variation in key fluconazole resistance genes
Source: Antimicrob Agents Chemother. 2024 May 7;68(6):e01619-23. doi: 10.1128/aac.01619-23 (PMC11620501; doi:10.1128/aac.01619-23)
Supplement: Fig. S3 — Genome-wide coverage analysis of selected isolates. [file aac.01619-23-s0004.pdf]

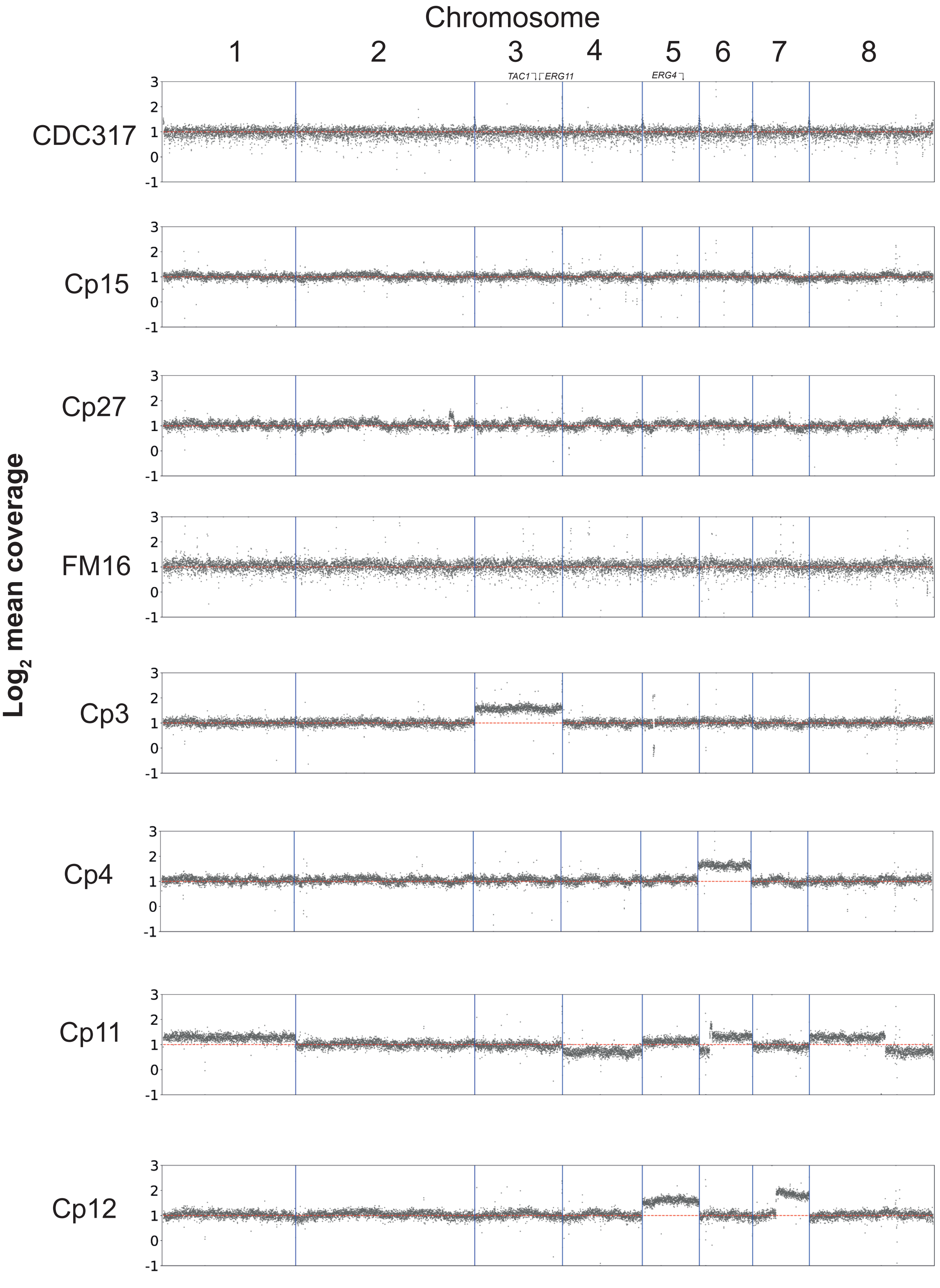

Chromosome
1
2
3
4
5
6
7
8
TAC1
ERG11
ERG4
CDC317
Cp15
Cp27
FM16
Cp3
Cp4
Cp11
Cp12
Log2 mean coverage
3
2
1
0
-1

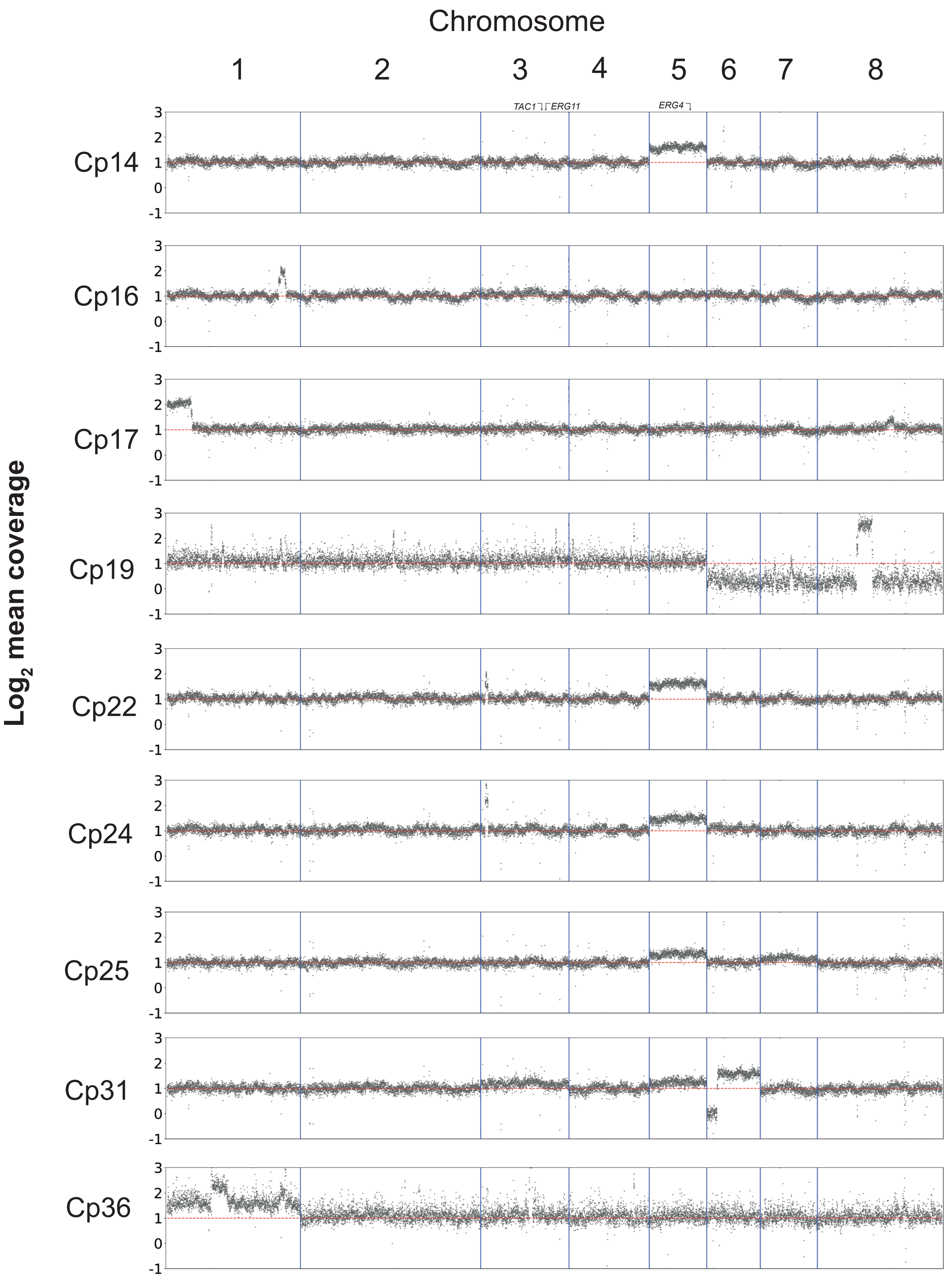

Chromosome
1
2
3
4
5
6
7
8
TAC1
ERG11
ERG4
Log2 mean coverage
Cp14
Cp16
Cp17
Cp19
Cp22
Cp24
Cp25
Cp31
Cp36
3
2
1
0
-1

Figure S3.

Genome-wide coverage tracks for strains of interest, including aneuploid strains. Coverage was calculated as the $\log_2$ mean coverage of 1 kb windows and plotted. Dashed red line shows $\log_2$ value = 1, i.e. typical 2x diploid coverage. The location of genes *TAC1*, *ERG11*, and *ERG4* are indicated with arrows.
