# Supplementary material for: Analysis of clinical Candida parapsilosis isolates reveals copy number variation in key fluconazole resistance genes
Source: Antimicrob Agents Chemother. 2024 May 7;68(6):e01619-23. doi: 10.1128/aac.01619-23 (PMC11620501; doi:10.1128/aac.01619-23)
Supplement: Table S3 — Source of sequencing data for isolates. [file aac.01619-23-s0007.docx]

**Table S3.** Source of sequencing data for isolates.

| **Isolate name** | **City** | **Country** | **Sequence data source** | **Reference** |
| --- | --- | --- | --- | --- |
| **Candida parapsilosis** | | | | |
| CP1 | Bloemfontein | South Africa | SRR28395763 | This study |
| CP10 | Bloemfontein | South Africa | SRR28395746 | This study |
| CP11 | Bloemfontein | South Africa | SRR28395747 | This study |
| CP12 | Bloemfontein | South Africa | SRR28395745 | This study |
| CP13 | New York | United States | SRR28395743 | This study |
| CP14 | Helsinki | Finland | SRR28395742,  [SRR28272126](https://trace.ncbi.nlm.nih.gov/Traces?run=SRR28272126) | This study,  This study (MinION) |
| CP15 | Quito | Ecuador | SRR28395741 | This study |
| CP16 | Bloemfontein | South Africa | SRR28395744 | This study |
| CP17 | Bloemfontein | South Africa | SRR28395740 | This study |
| CP18 | Bloemfontein | South Africa | SRR28395739 | This study |
| CP19 | Bloemfontein | South Africa | SRR28395738 | This study |
| CP2 | Bloemfontein | South Africa | SRR28395762 | This study |
| CP20 | Bloemfontein | South Africa | SRR28395737 | This study |
| CP21 | Ann Arbor | United States | SRR28395734 | This study |
| CP22 | Johannesburg | South Africa | SRR28395733 | This study |
| CP24 | Johannesburg | South Africa | SRR28395732 | This study |
| CP25 | Johannesburg | South Africa | SRR28395731 | This study |
| CP26 | Johannesburg | South Africa | SRR28395730 | This study |
| CP27 | Hershey | United States | SRR28395729 | This study |
| CP28 | Bloemfontein | South Africa | SRR28395727 | This study |
| CP29 | Bloemfontein | South Africa | SRR28395726 | This study |
| CP3 | Kuala Lumpur | Malaysia | SRR28395735 | This study |
| CP30 | Bratislava | Slovakia | SRR28395725 | This study |
| CP31 | Johannesburg | South Africa | SRR28395761 | This study |
| CP32 | Caracas | Venezuela | SRR28395760 | This study |
| CP34 | Johannesburg | South Africa | SRR28395759 | This study |
| CP35 | Johannesburg | South Africa | SRR28395758 | This study |
| CP36 | Detroit | United States | SRR28395757 | This study |
| CP37 | Johannesburg | South Africa | SRR28395756 | This study |
| CP38 | Johannesburg | South Africa | SRR28395755 | This study |
| CP39 | Bratislava | Slovakia | SRR28395754 | This study |
| CP4 | Johannesburg | South Africa | SRR28395728 | This study |
| CP5 | New York | United States | SRR28395752 | This study |
| CP6 | Turino | Italy | SRR28395751 | This study |
| CP7 | Johannesburg | South Africa | SRR28395750 | This study |
| CP8 | Bloemfontein | South Africa | SRR28395749 | This study |
| CP9 | Bloemfontein | South Africa | SRR28395748 | This study |
| CDC317 | Mississippi | USA | SRR17522876,  [SRR28272127](https://trace.ncbi.nlm.nih.gov/Traces?run=SRR28272127) | (Butler et al, 2009)_1_,  This study (MinION) |
| FM16 | Nantes | France | SRR17522859 | (Bergin et al, 2022)_2_ |
| MSK809 | New York | USA | SRR17231046 | (Bergin et al, 2022)_2_ |
| 73-037 | Leeds | UK | SRR17522867 | (Bergin et al, 2022)_2_ |
| 73-114 | Leeds | UK | SRR17522850 | (Bergin et al, 2022)_2_ |
| **Candida orthopsilosis** | | | | |
| 151 | Atlanta | USA | SRR3547357 | (Schröder et al, 2016)_3_ |
| 185 | Atlanta | USA | SRR3547479 | (Schröder et al, 2016)_3_ |
| 282 | Baltimore | USA | SRR3547496 | (Schröder et al, 2016)_3_ |
| 320 | Baltimore | USA | SRR3547558 | (Schröder et al, 2016)_3_ |
| 421 | Pisa | Italy | SRR3547554 | (Schröder et al, 2016)_3_ |
| 422 | Pisa | Italy | SRR3547557 | (Schröder et al, 2016)_3_ |
| 423 | L'Aquila | Italy | SRR3547559 | (Schröder et al, 2016)_3_ |
| 424 | Pisa | Italy | SRR3547561 | (Schröder et al, 2016)_3_ |
| 425 | Pisa | Italy | SRR3547626 | (Schröder et al, 2016)_3_ |
| 426 | Varese | Italy | SRR3547627 | (Schröder et al, 2016)_3_ |
| 427 | Pisa | Italy | SRR3547628 | (Schröder et al, 2016)_3_ |
| 428 | Hong Kong | Hong Kong | SRR3547629 | (Schröder et al, 2016)_3_ |
| 433 | Sint-Niklaas | Belgium | SRR3547630 | (Schröder et al, 2016)_3_ |
| 434 | Bristol | UK | SRR3547632 | (Schröder et al, 2016)_3_ |
| 435 | Pisa | Italy | SRR3547633 | (Schröder et al, 2016)_3_ |
| 436 | Pisa | Italy | SRR3547635 | (Schröder et al, 2016)_3_ |
| 437 | Pisa | Italy | SRR3547653 | (Schröder et al, 2016)_3_ |
| 498 | Baltimore | USA | SRR3547678 | (Schröder et al, 2016)_3_ |
| 504 | Baltimore | USA | SRR3547701 | (Schröder et al, 2016)_3_ |
| 599 | Baltimore | USA | SRR3547726 | (Schröder et al, 2016)_3_ |
| 748 | Atlanta | USA | SRR3547806 | (Schröder et al, 2016)_3_ |
| 831 | Atlanta | USA | SRR3547961 | (Schröder et al, 2016)_3_ |
| 1540 | Baltimore | USA | SRR3547359 | (Schröder et al, 2016)_3_ |
| 1799 | Atlanta | USA | SRR3547474 | (Schröder et al, 2016)_3_ |
| 1825 | Baltimore | USA | SRR3547478 | (Schröder et al, 2016)_3_ |
| 90-125 | N/A | N/A | SRR6509076 | (Schröder et al, 2016)_3_ |
| B-8274 | N/A | Pakistan | SRR3547962 | (Schröder et al, 2016)_3_ |
| B-8323 | N/A | Pakistan | SRR3547965 | (Schröder et al, 2016)_3_ |
| MCO456 | N/A | N/A | ERR321926 | (Pryszcz et al, 2014)_4_ |
| MSK477 | New York | USA | SRR10335747 | (Zhai et al, 2020)_5_ |
| MSK479 | New York | USA | SRR10335746 | (Zhai et al, 2020)_5_ |
| MSK616 | New York | USA | SRR17231074 | (Bergin et al, 2022) _2_ |
| MSK636 | New York | USA | SRR10335750 | (Zhai et al, 2020)_5_ |
| MSK638 | New York | USA | SRR10335749 | (Zhai et al, 2020)_5_ |
| MSK639 | New York | USA | SRR10335748 | (Zhai et al, 2020)_5_ |
| MSK805 | New York | USA | SRR17231065 | (Bergin et al, 2022)_2_ |
| **Candida metapsilosis** | | | | |
| MSK403 | New York | USA | SRR10335720 | (Zhai et al, 2020)_5_ |
| MSK404 | New York | USA | SRR10335718 | (Zhai et al, 2020)_5_ |
| MSK413 | New York | USA | SRR10335745 | (Zhai et al, 2020)_5_ |
| MSK414 | New York | USA | SRR15193271 | (O’Brien et al, 2022)_6_ |
| MSK415 | New York | USA | SRR10335744 | (Zhai et al, 2020)_5_ |
| MSK416 | New York | USA | SRR10335743 | (Zhai et al, 2020)_5_ |
| MSK417 | New York | USA | SRR10335742 | (Zhai et al, 2020)_5_ |
| MSK418 | New York | USA | SRR10335740 | (Zhai et al, 2020)_5_ |
| MSK429 | New York | USA | SRR10335739 | (Zhai et al, 2020)_5_ |
| MSK430 | New York | USA | SRR10335738 | (Zhai et al, 2020)_5_ |
| MSK431 | New York | USA | SRR10335737 | (Zhai et al, 2020)_5_ |
| MSK432 | New York | USA | SRR10335736 | (Zhai et al, 2020)_5_ |
| MSK433 | New York | USA | SRR10335735 | (Zhai et al, 2020)_5_ |
| MSK434 | New York | USA | SRR10335734 | (Zhai et al, 2020)_5_ |
| MSK445 | New York | USA | SRR10335733 | (Zhai et al, 2020)_5_ |
| MSK446 | New York | USA | SRR10335732 | (Zhai et al, 2020)_5_ |
| MSK447 | New York | USA | SRR10335731 | (Zhai et al, 2020)_5_ |
| MSK448 | New York | USA | SRR10335729 | (Zhai et al, 2020)_5_ |
| MSK449 | New York | USA | SRR10335728 | (Zhai et al, 2020)_5_ |
| MSK450 | New York | USA | SRR10335727 | (Zhai et al, 2020)_5_ |
| MSK461 | New York | USA | SRR10335726 | (Zhai et al, 2020)_5_ |
| MSK462 | New York | USA | SRR10335725 | (Zhai et al, 2020)_5_ |
| MSK463 | New York | USA | SRR10335724 | (Zhai et al, 2020)_5_ |
| MSK464 | New York | USA | SRR10335723 | (Zhai et al, 2020)_5_ |
| MSK465 | New York | USA | SRR10335722 | (Zhai et al, 2020)_5_ |
| MSK466 | New York | USA | SRR10335721 | (Zhai et al, 2020)_5_ |
| MSK606 | New York | USA | SRR15193270 | (O’Brien et al, 2022)_6_ |
| MSK607 | New York | USA | SRR15193269 | (O’Brien et al, 2022)_6_ |
| MSK798 | New York | USA | SRR15193268 | (O’Brien et al, 2022)_6_ |
| MSK801 | New York | USA | SRR15193267 | (O’Brien et al, 2022)_6_ |

**References**

1. Butler G, Rasmussen MD, Lin MF, Santos MA, Sakthikumar S, Munro CA, Rheinbay E, Grabherr M, Forche A, Reedy JL. 2009. Evolution of pathogenicity and sexual reproduction in eight Candida genomes. Nature 459:657-662.

2. Bergin SA, Zhao F, Ryan AP, Müller CA, Nieduszynski CA, Zhai B, Rolling T, Hohl TM, Morio F, Scully J. 2022. Systematic analysis of copy number variations in the pathogenic yeast Candida parapsilosis identifies a gene amplification in RTA3 that is associated with drug resistance. Mbio 13:e01777-22.

3. Schröder MS, Martinez de San Vicente K, Prandini TH, Hammel S, Higgins DG, Bagagli E, Wolfe KH, Butler G. 2016. Multiple origins of the pathogenic yeast Candida orthopsilosis by separate hybridizations between two parental species. PLoS genetics 12:e1006404.

4. Pryszcz LP, Nemeth T, Gacser A, Gabaldon T. 2014. Genome comparison of Candida orthopsilosis clinical strains reveals the existence of hybrids between two distinct subspecies. Genome biology and evolution 6:1069-1078.

5. Zhai B, Ola M, Rolling T, Tosini NL, Joshowitz S, Littmann ER, Amoretti LA, Fontana E, Wright RJ, Miranda E. 2020. High-resolution mycobiota analysis reveals dynamic intestinal translocation preceding invasive candidiasis. Nature medicine 26:59-64.

6. O’Brien CE, Zhai B, Ola M, Bergin SA, Ó Cinnéide E, O’Connor Í, Rolling T, Miranda E, Babady NE, Hohl TM. 2022. Identification of a novel Candida metapsilosis isolate reveals multiple hybridization events. G3 12:jkab367.
